# A nationwide survey of infection prevention and control and high-level disinfection and sterilization practices in the Dominican Republic

**DOI:** 10.1017/ash.2026.10788

**Published:** 2026-07-06

**Authors:** Elianet Castillo, Rita Rojas-Fermin, Claudia Blanco, Antonio Villegas, Yeison Reyes, Alfredo J. Mena Lora

**Affiliations:** 1 CEDIMAT: Centros de Diagnostico y Medicina Avanzada y de Conferencias Medicas y, Dominican Republic; 2 Hospital General Plaza de la Salud, Santo Domingo, Dominican Republic; 3 Department of Medicine, University of Illinois Chicago, Chicago, USA

## Abstract

Healthcare-associated infections (HAIs) are often preventable and cause significant morbidity and mortality. There is a paucity of data on HAIs in low- and middle-income countries. This study surveyed Dominican Republic hospitals, revealing that 60% had Infection Prevention, and Control (IPC) programs, but gaps remain in IPC committees, training, data tracking, and reporting.

## Introduction

Hospital-acquired infections (HAIs) are a significant global health challenge, affecting patients in both developed and developing countries. They are often preventable and are associated with high morbidity and mortality, prolonged hospital stays, increased microbial resistance to antimicrobials, and substantial financial burdens on patients, families, and healthcare systems. In low- and middle-income countries (LMICs), approximately 15% of patients in acute care hospitals acquire an HAI, compared to 7% in high-income countries.^1^

Despite the global burden of HAIs, many countries lack robust Infection Prevention and Control (IPC) programs. Among 162 countries submitting data to the World Health Organization (WHO), 11% reported having no IPC program or operational plan, while 54% indicated that their IPC programs were either not implemented or limited to selected facilities.^2^ Only 34% of countries reported nationwide implementation of IPC programs, and of these, just 19% had systems to monitor effectiveness and compliance.^2^ However, evidence suggests that most HAIs are preventable with effective IPC measures capable of reducing infection rates by up to 70%.^3^

Despite this potential, little is known about the implementation of IPC programs in Latin America and the Caribbean, particularly regarding critical areas such as high-level disinfection and sterilization practices. Our study aims to evaluate the status of IPC programs in the Dominican Republic (DR), with a specific focus on high-level disinfection and sterilization practices, providing much-needed insights into regional infection prevention efforts.

## Methods

### Study design and participants

We conducted a cross-sectional survey of infectious diseases (ID) specialists working in acute care hospitals in the Dominican Republic (DR) to assess IPC practices, with a specific focus on high-level disinfection and sterilization. A convenience sample of 20 acute care hospitals was selected to include hospitals from multiple geographic regions and a range of healthcare settings within the Dominican Republic. One ID physician representing each hospital was invited to participate. Responses were received from all hospitals contacted. The survey was adapted from two Centers for Disease Control and Prevention Infection Control Assessment and Response (ICAR) tools: the general ICAR tool for IPC across healthcare settings and the ICAR tool for high-level disinfection and sterilization (Supplement 1). The ICAR framework was selected for its detailed assessment of device reprocessing, sterilization, and HLD practices and broad IPC program elements. Respondents were asked to provide anonymous information about facility demographics, critical infrastructure, and policies, procedures, and practices related to medical device reprocessing.

### Data analysis

Data were tabulated, and descriptive statistical analyses were performed. The ICAR tool includes a comprehensive set of questions designed to evaluate facility demographics, infrastructure, and medical device reprocessing policies and practices. This structured approach allowed for a systematic assessment of IPC measures implemented in healthcare facilities.

### Ethics

This study was approved by the Ethical Investigation Committee of CEDIMAT in March 2024.

## Results

**Figure 1.**
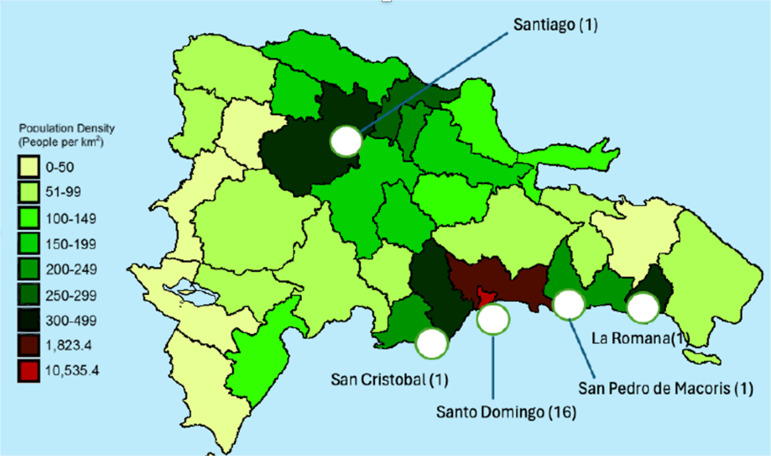
Population density of the Dominican Republic and geographic distribution of hospitals surveyed.

**Figure 2.**
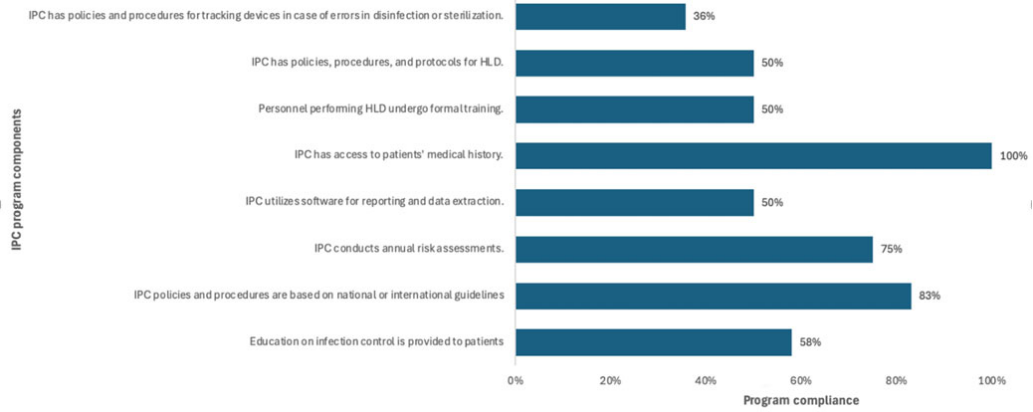
Components of infection prevention and control programs in the Dominican Republic.

A total of 20 hospitals responded to the survey, with 16 facilities located in Santo Domingo and one in Santiago, San Cristobal, La Romana, and San Pedro de Macoris (Figure 1). Among the surveyed facilities, 60% (12/20) reported having an IPC program, 55% (11/20) had a dedicated multidisciplinary IPC committee, and 90% (18/20) had access to an IPC expert, such as an ID physician (Figure 2).

Patient education on IPC measures was provided in 58% (11/20) of hospitals. Additionally, 83% (15/20) of facilities set policies and procedures based on national or international guidelines, 75% (15/20) conducted annual risk assessments, and 50% (10/20) utilized software for data reporting and extraction. All facilities with IPC programs reported having access to medical records for IPC-related activities.

Fourteen hospitals provided specific data on HLD and sterilization practices. Among these, only 50% (7/14) reported that staff performing HLD and sterilization were formally trained, and 50% (7/14) had specific policies, procedures, and protocols in place for these processes. Reuse of single-use devices was reported by 57.1% (8/14) of respondents, 35.7% (5/14) tracked devices for errors, and 28.5% (4/14) had a reporting process for device-related infections to public health authorities.

## Discussion

Our survey provides a comprehensive overview of IPC practices in DR hospitals, highlighting areas of improvement for program implementation and adherence to key IPC components. Forty percent of surveyed hospitals reported no IPC program, and among hospitals with IPC programs, gaps remained in committee structure, training, data tracking systems, and reporting mechanisms.

Similar gaps exist in other LMICs in Latin America. A 2024 study assessing IPC implementation in 37 healthcare facilities across Argentina, Ecuador, Guatemala, and Panama using the WHO Infection Prevention and Control Assessment Framework reported a median score of 614, indicating advanced IPC implementation.^4^ While guideline adoption was widespread (95%), key deficiencies included inadequate staffing, education, and multimodal strategies. Similarly, in our survey, 83% of hospitals reported policies based on national or international standards, but only 55% had dedicated IPC committees. Notably, 90% of hospitals in our study had access to IPC experts, exceeding the 30% staffing rate per 110 beds observed in the 2024 study.^4^ A 2021 study evaluating IPC practices in 267 Colombian hospitals reported a median IPC performance score of 672, with high scores for guidelines and infrastructure but low scores for staffing and education.^5^ These regional studies underscore the progress made in IPC across Latin America, although challenges in resource allocation and implementation persist.

Our survey also identified significant areas of improvement for HLD and sterilization practices. While 50% of surveyed hospitals reported having HLD and sterilization policies, compliance and training were inconsistent. Only half of the staff performing these procedures were formally trained, aligning with a 2017 Brazilian study where 56% of sterile processing department (SPD) staff received training within two years.^8^ Furthermore, 57.1% of hospitals in our survey reported reusing single-use devices, 35.7% tracked devices for errors, and 28.5% had a reporting process for device-related infections.

Regional studies corroborate these findings. In Peru, a 2014 study demonstrated compliance with disinfection and drying protocols but poor adherence to precleaning (30%) and cleaning (16.7%).^6^ Similarly, Argentinian and Brazilian studies reported significant gaps in bronchoscope reprocessing and SPD protocols, respectively.^7^

### Limitations

The use of a convenience sample may add selection bias and not fully represent all hospitals in the DR. The modified survey instrument may not capture all aspects of IPC implementation. Respondent characteristics such as years of experience and formal IPC training were not collected.

### Conclusions

Despite these limitations, our findings highlight substantial gaps in IPC program development, implementation, and training, particularly regarding HLD and sterilization. Strengthening these is crucial to aligning IPC measures with international standards. Future studies should focus on evaluating the impact of IPC programs on infection rates, healthcare costs, and antimicrobial resistance in hospitals.

## Supporting information

10.1017/ash.2026.10788.sm001 Castillo et al. supplementary material Castillo et al. supplementary material

## Supplementary material

The supplementary material for this article can be found at https://doi.org/10.1017/ash.2026.10788.
