# Supplementary material for: A nationwide survey of infection prevention and control and high-level disinfection and sterilization practices in the Dominican Republic
Source: Antimicrob Steward Healthc Epidemiol. 2026 Jul 6;6(1):e204. doi: 10.1017/ash.2026.10788 (PMC13343334; doi:10.1017/ash.2026.10788)

**Supplementary Appendix**

Supplement 1. Survey instrument used to assess infection prevention and control and high-level disinfection/sterilization practices in Dominican Republic hospitals.


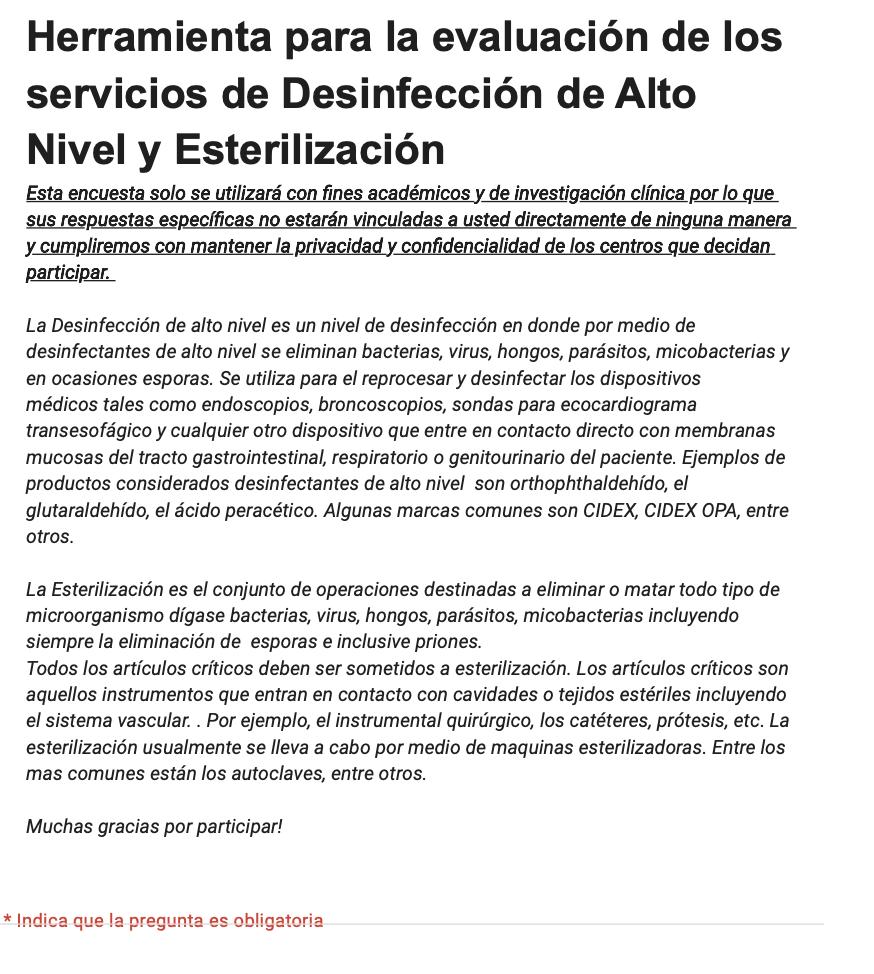


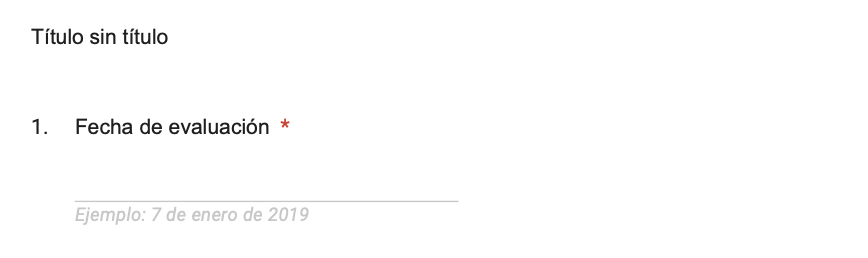


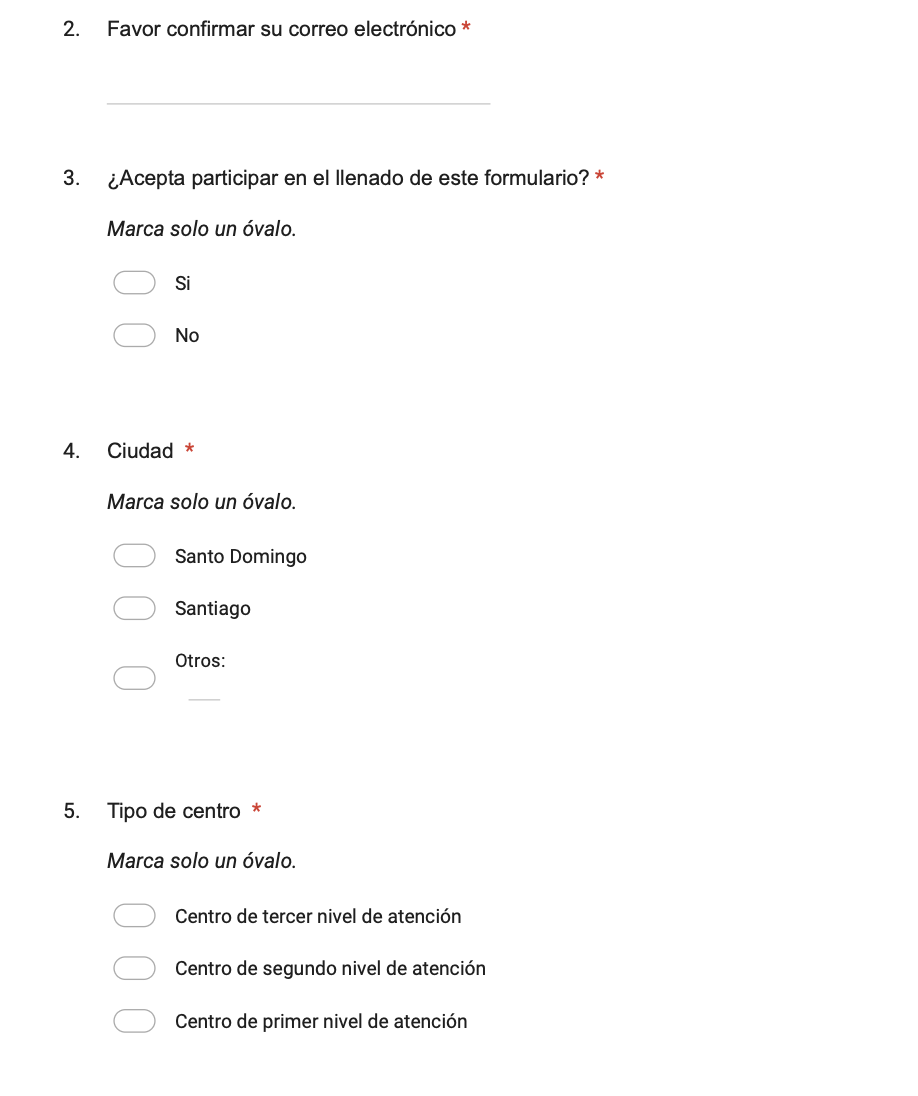


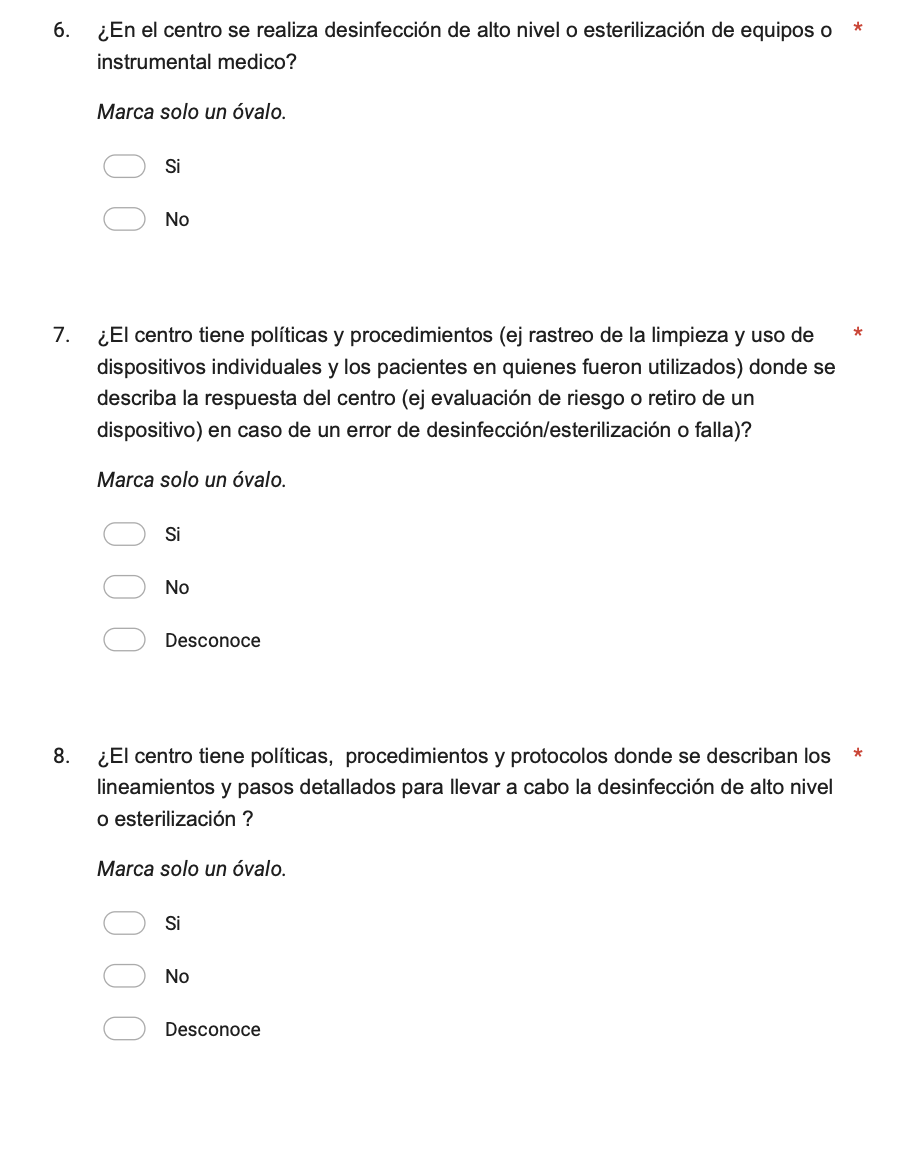

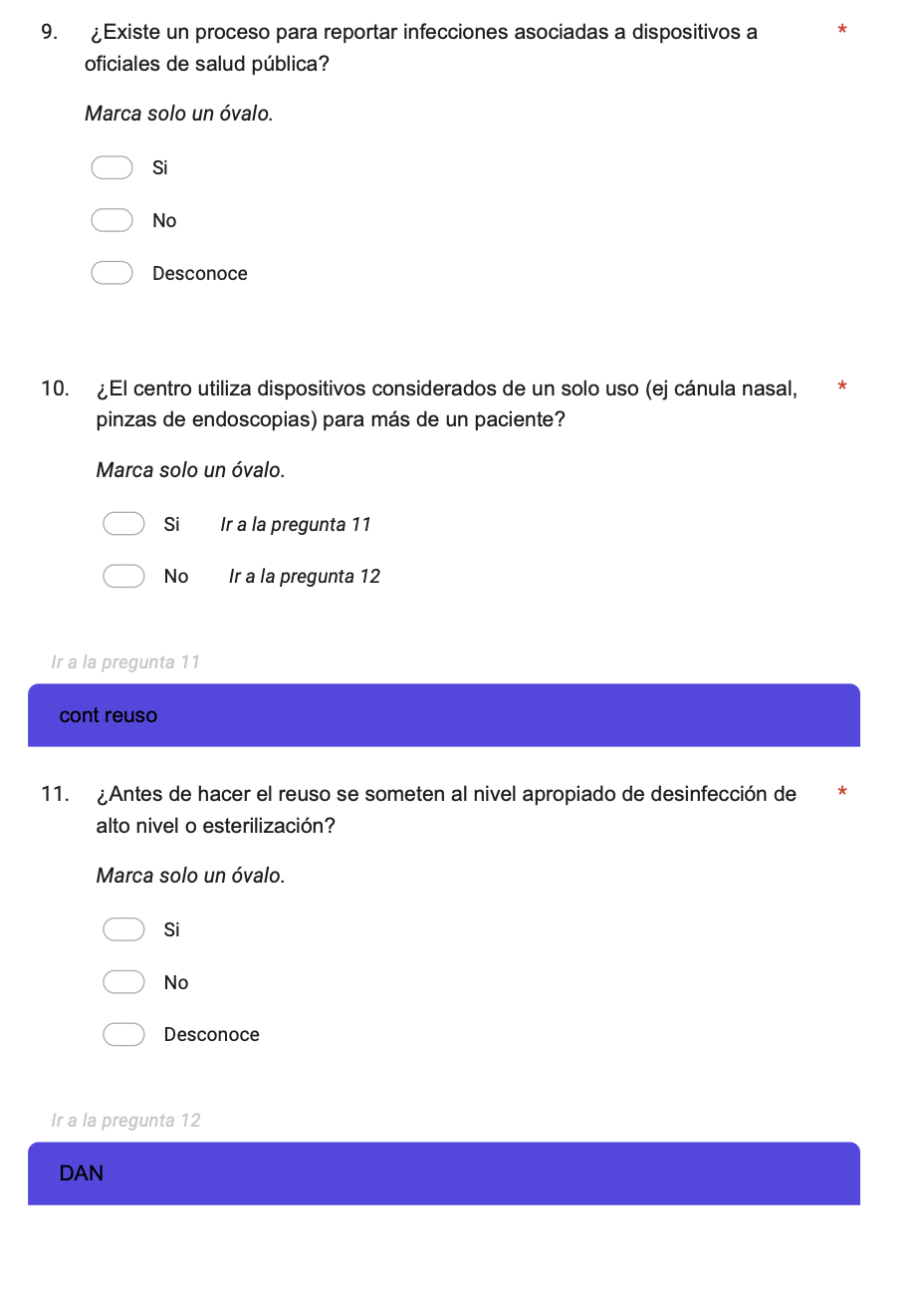


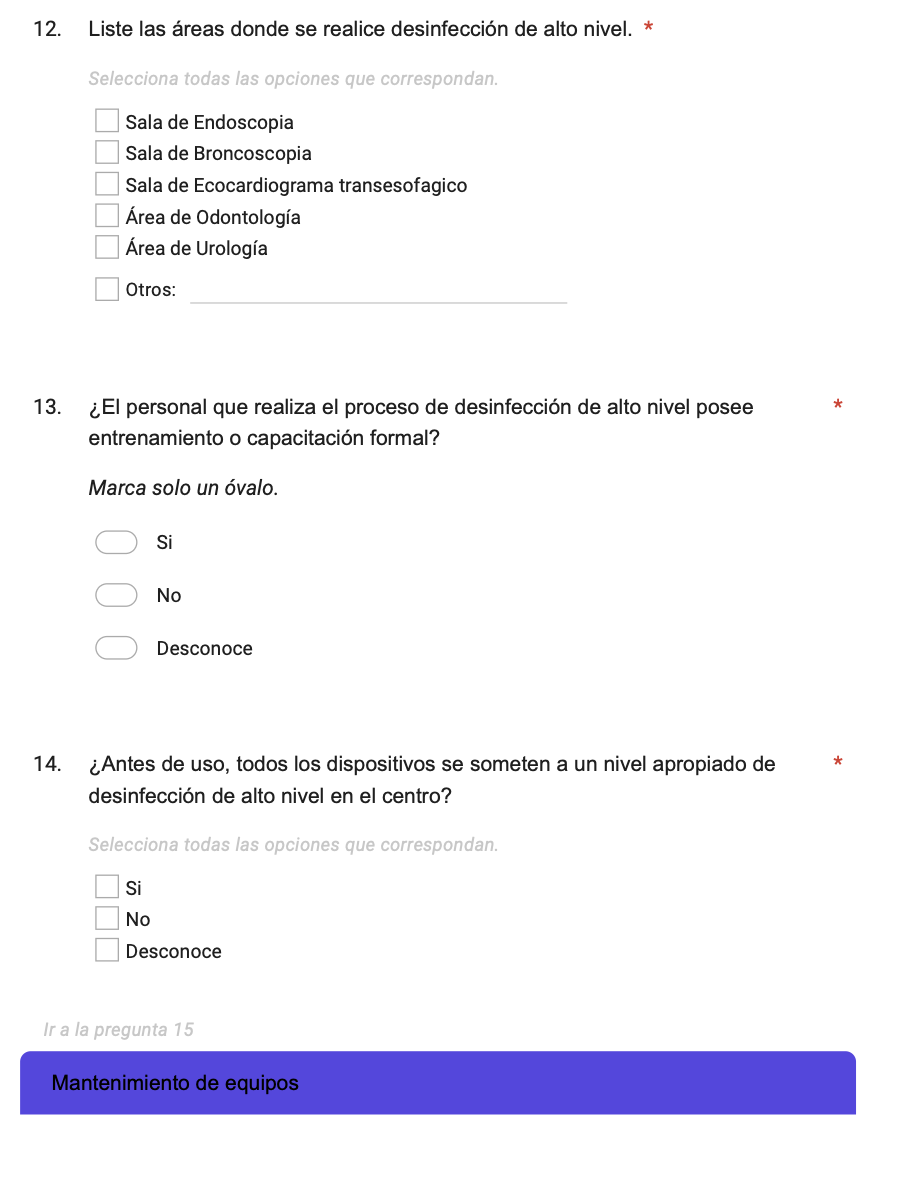


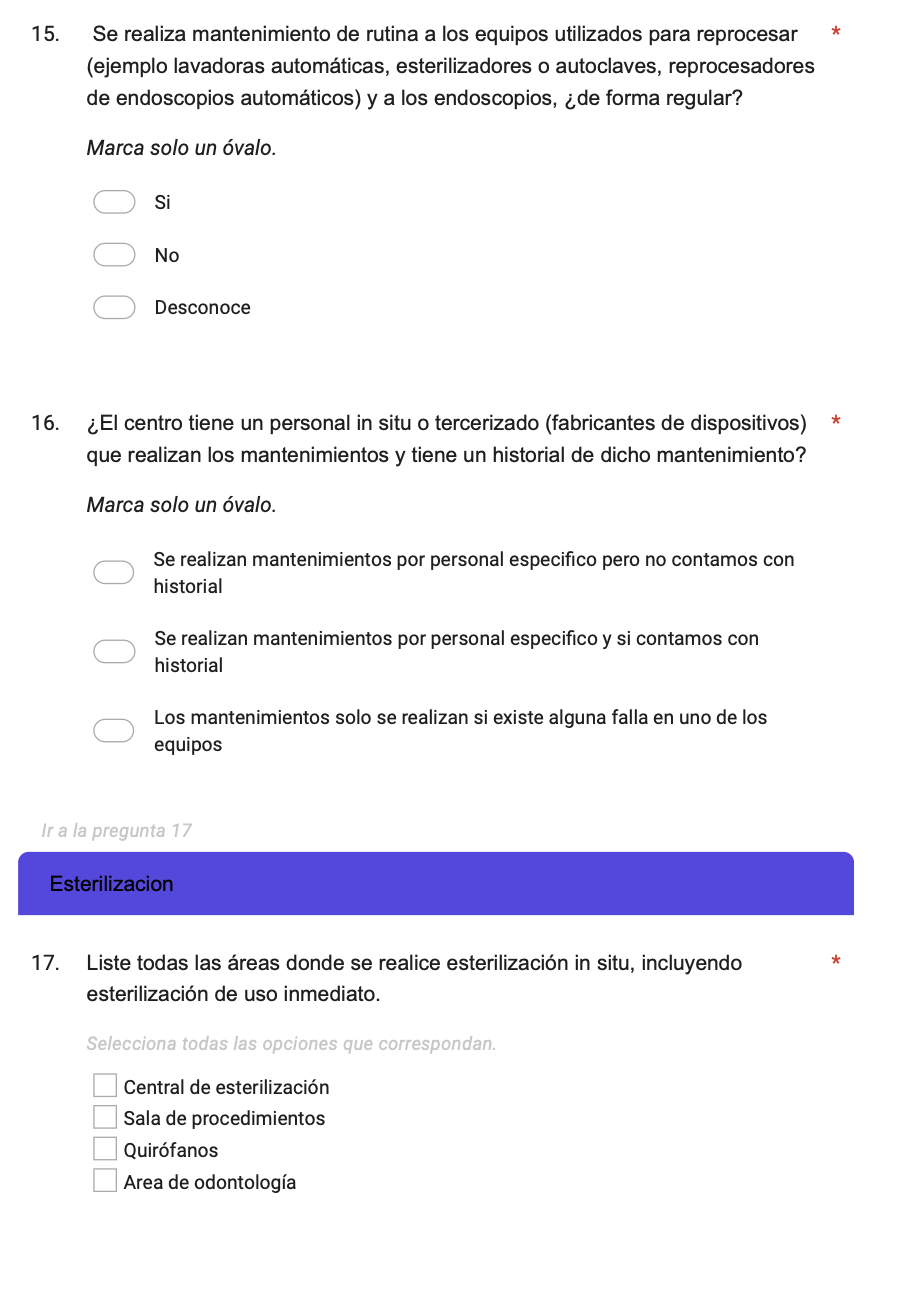

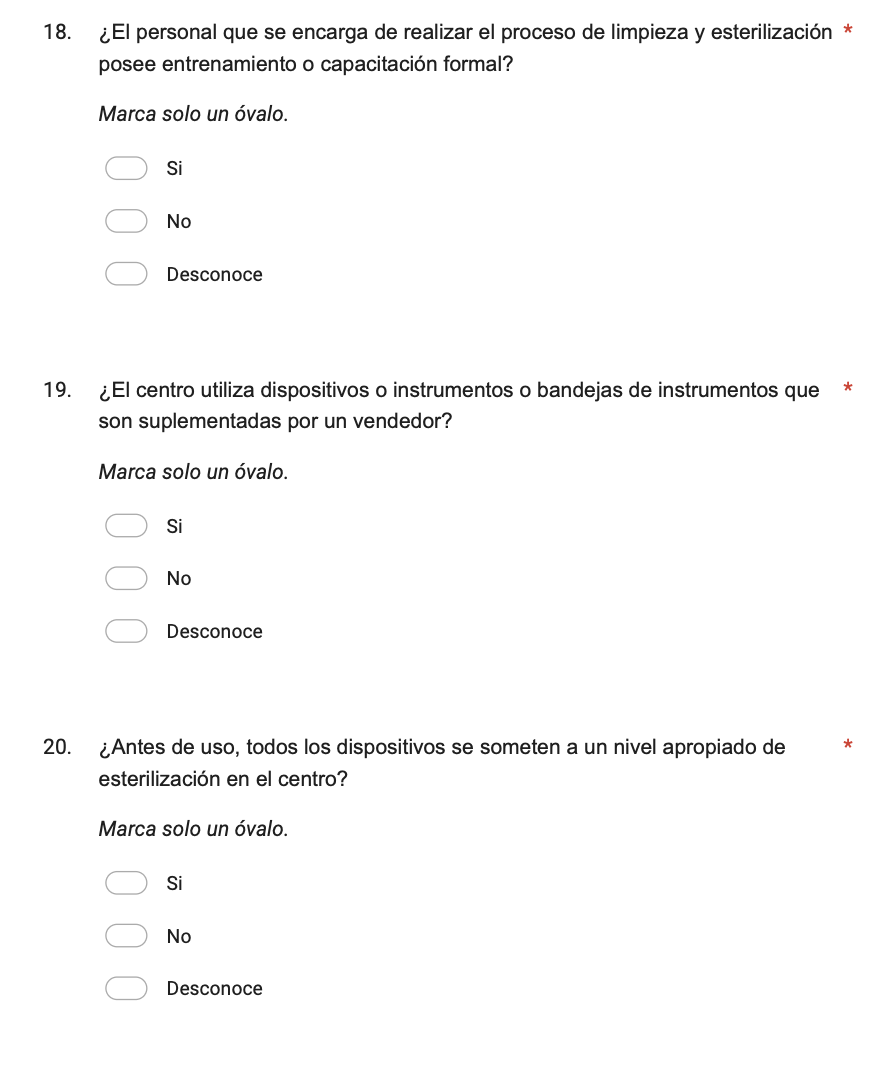


Supplement 2.


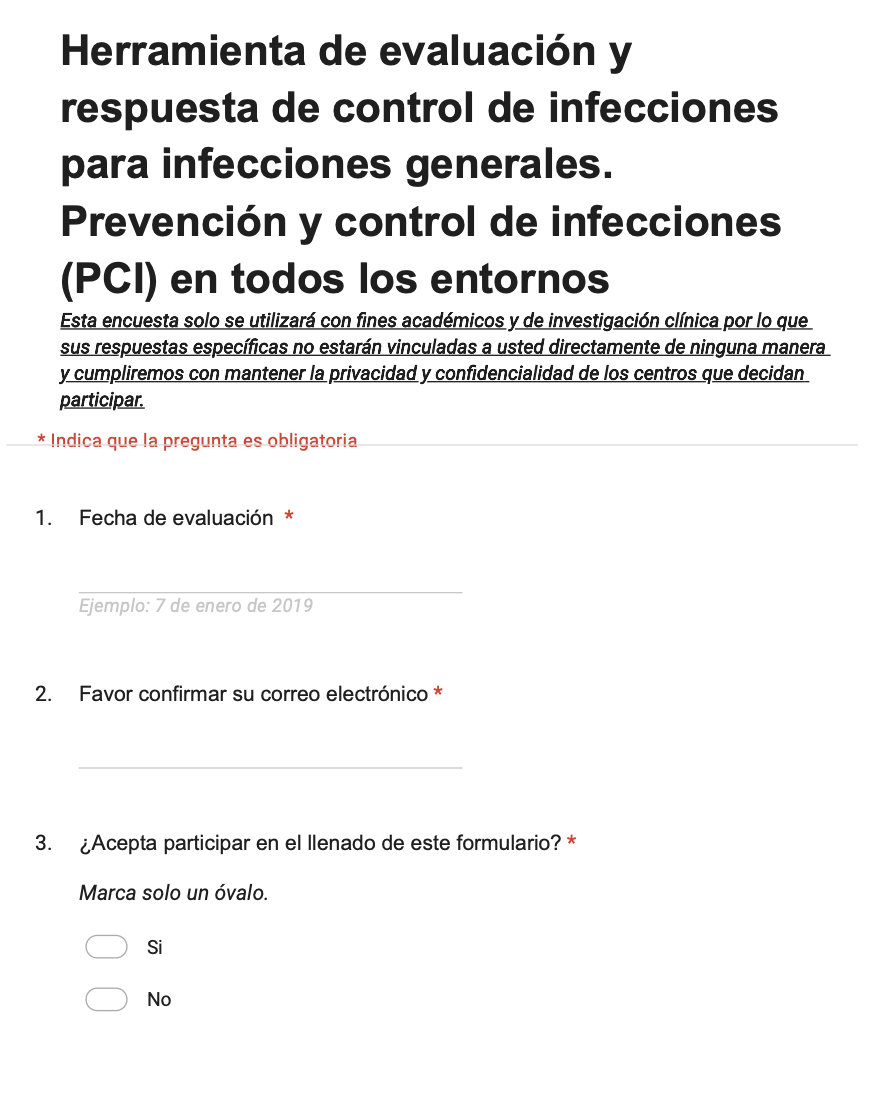


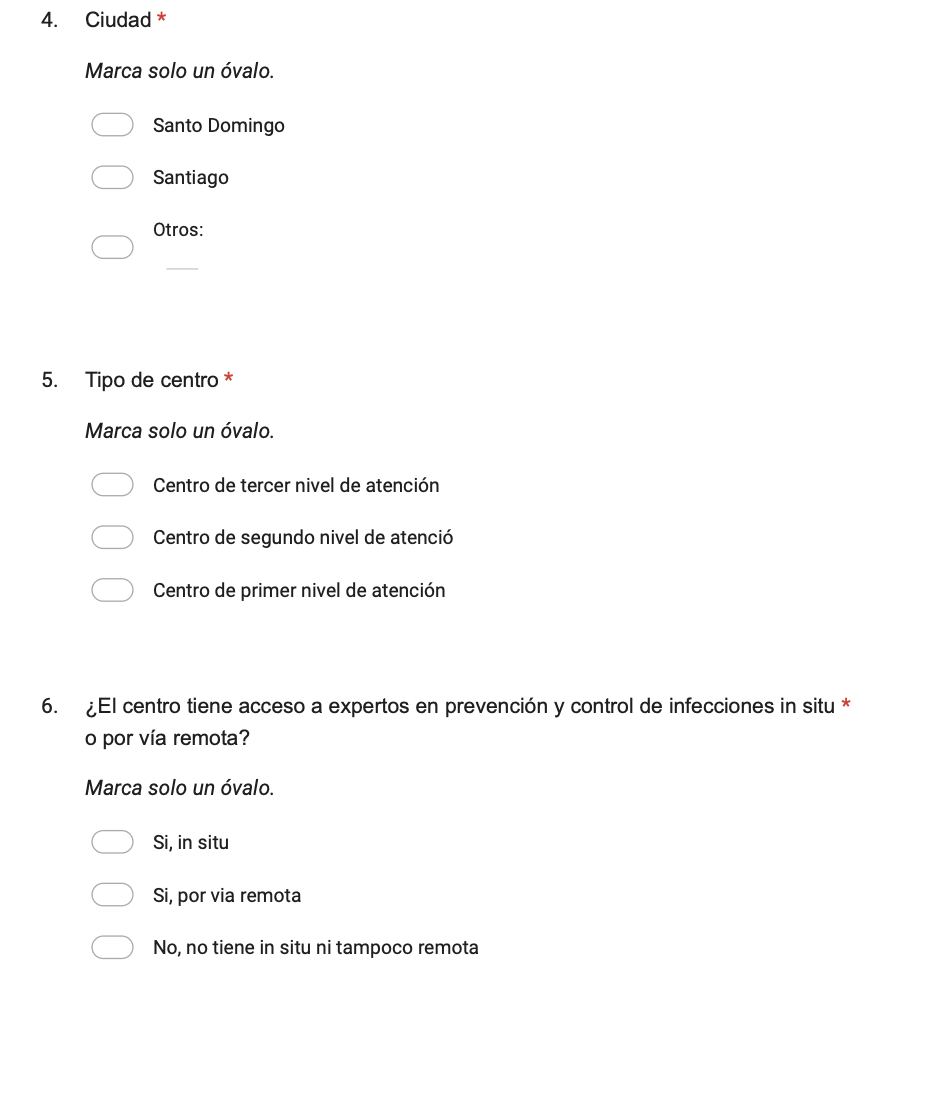

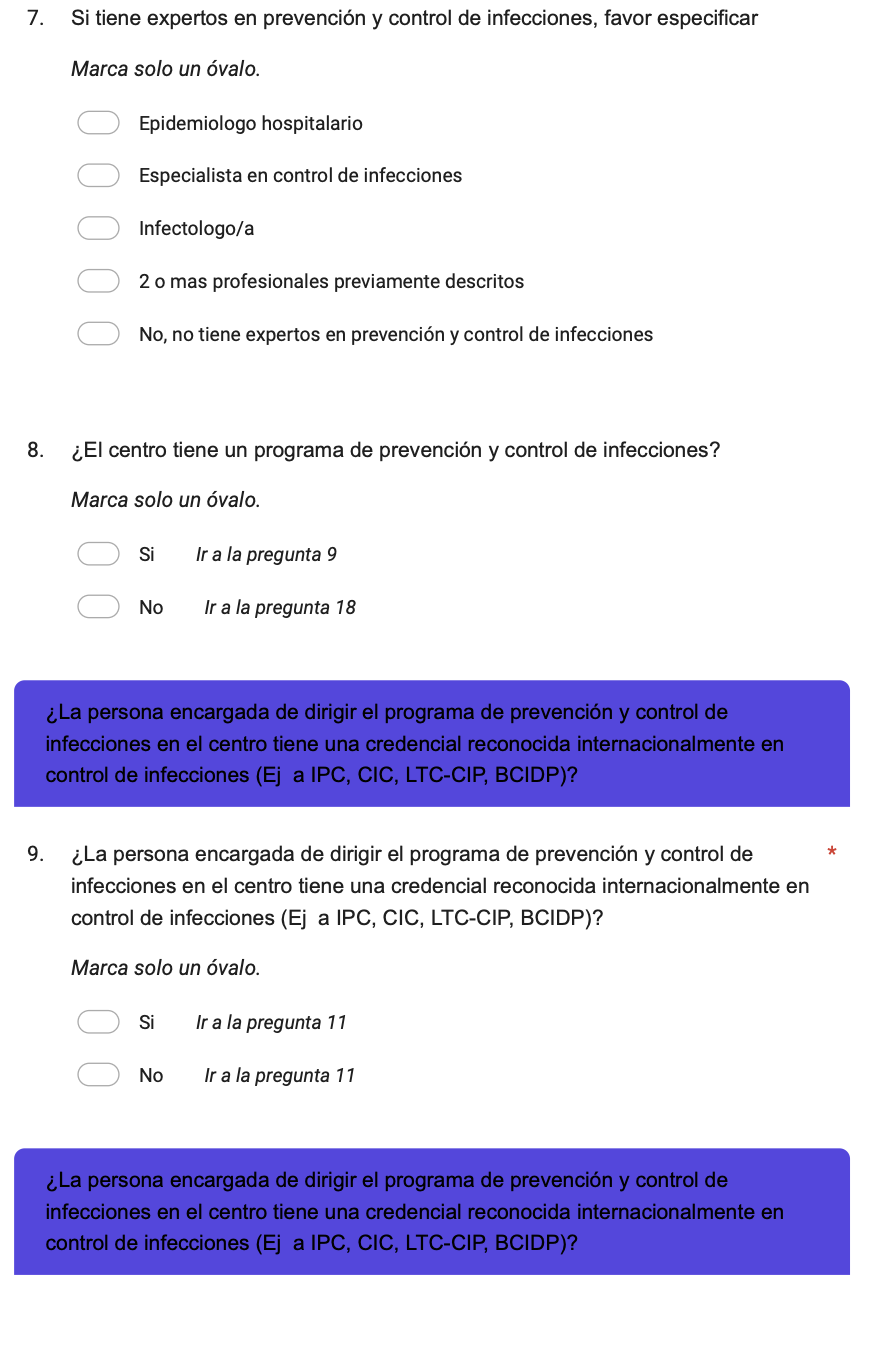


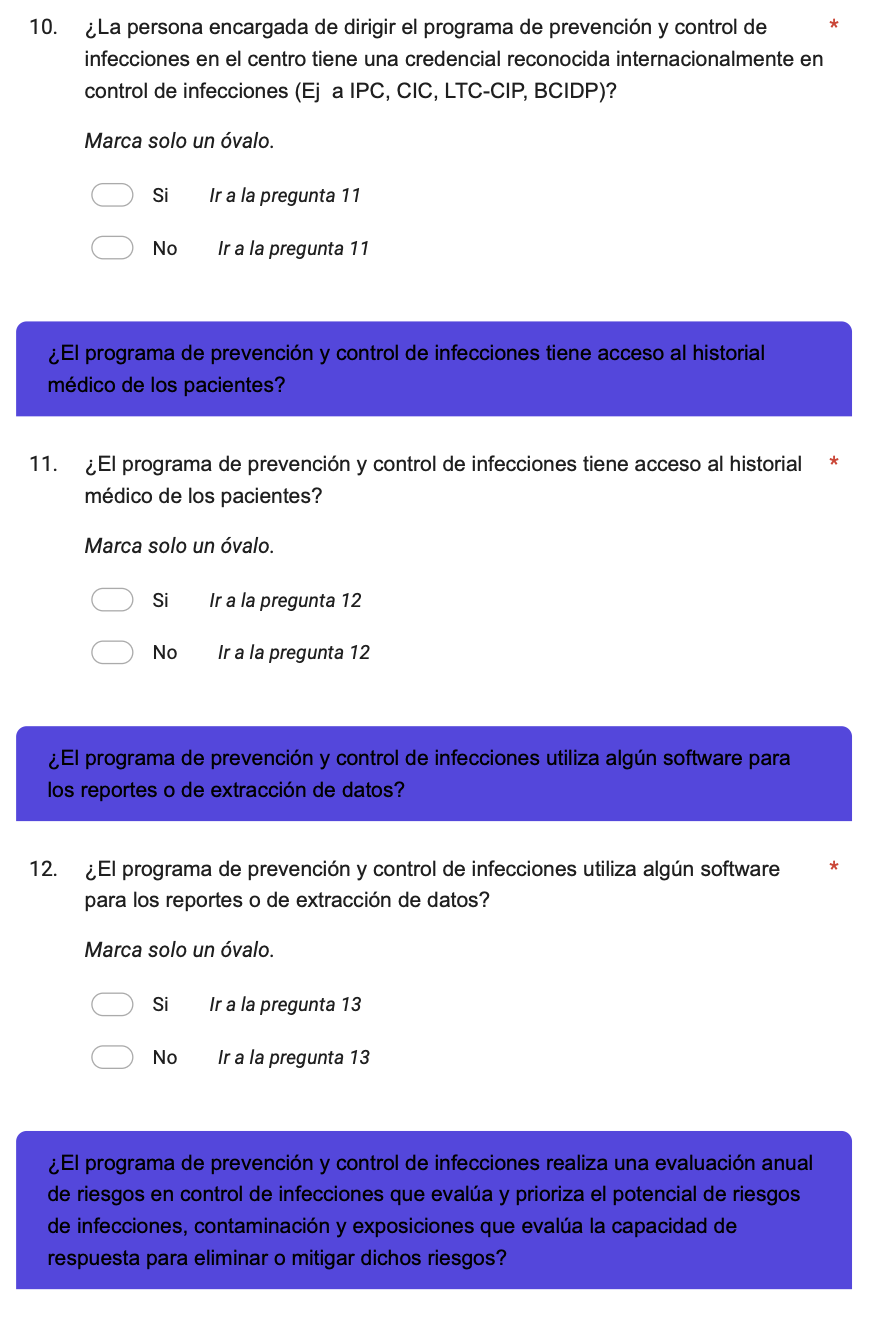

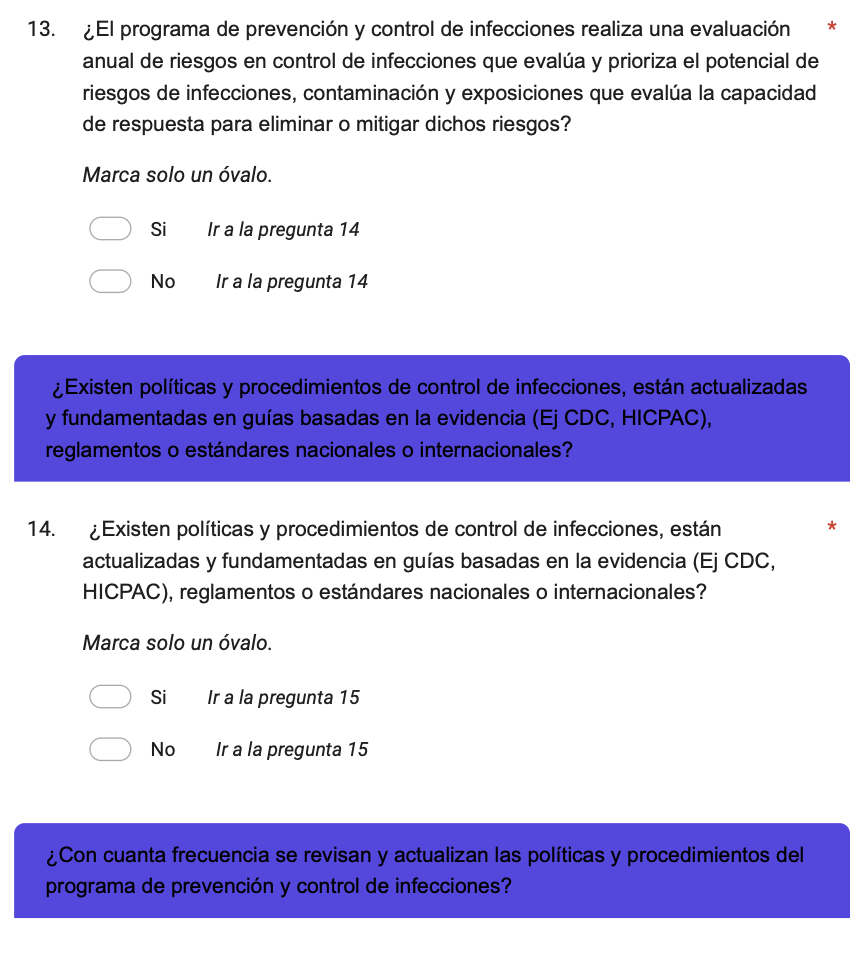

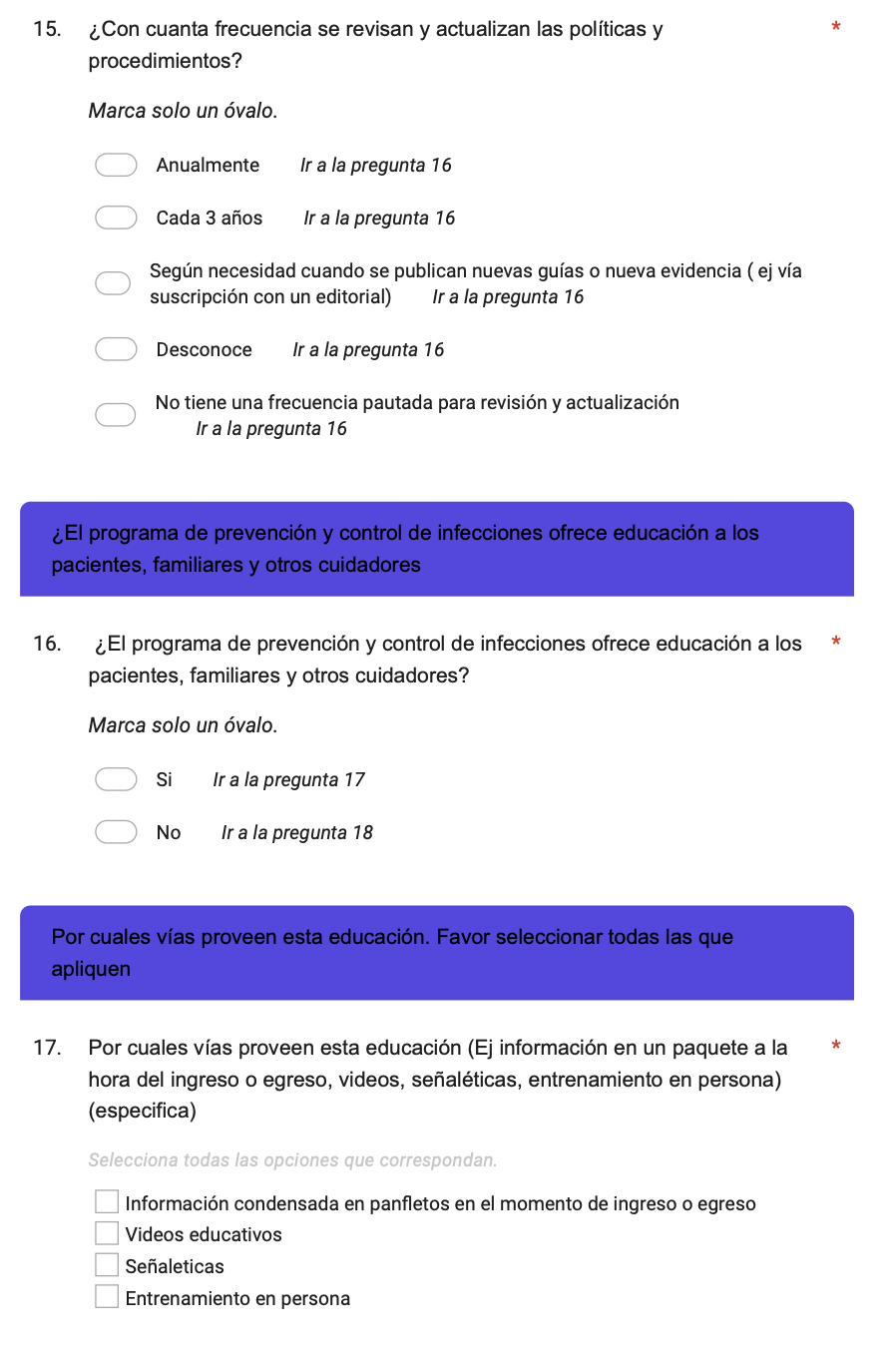

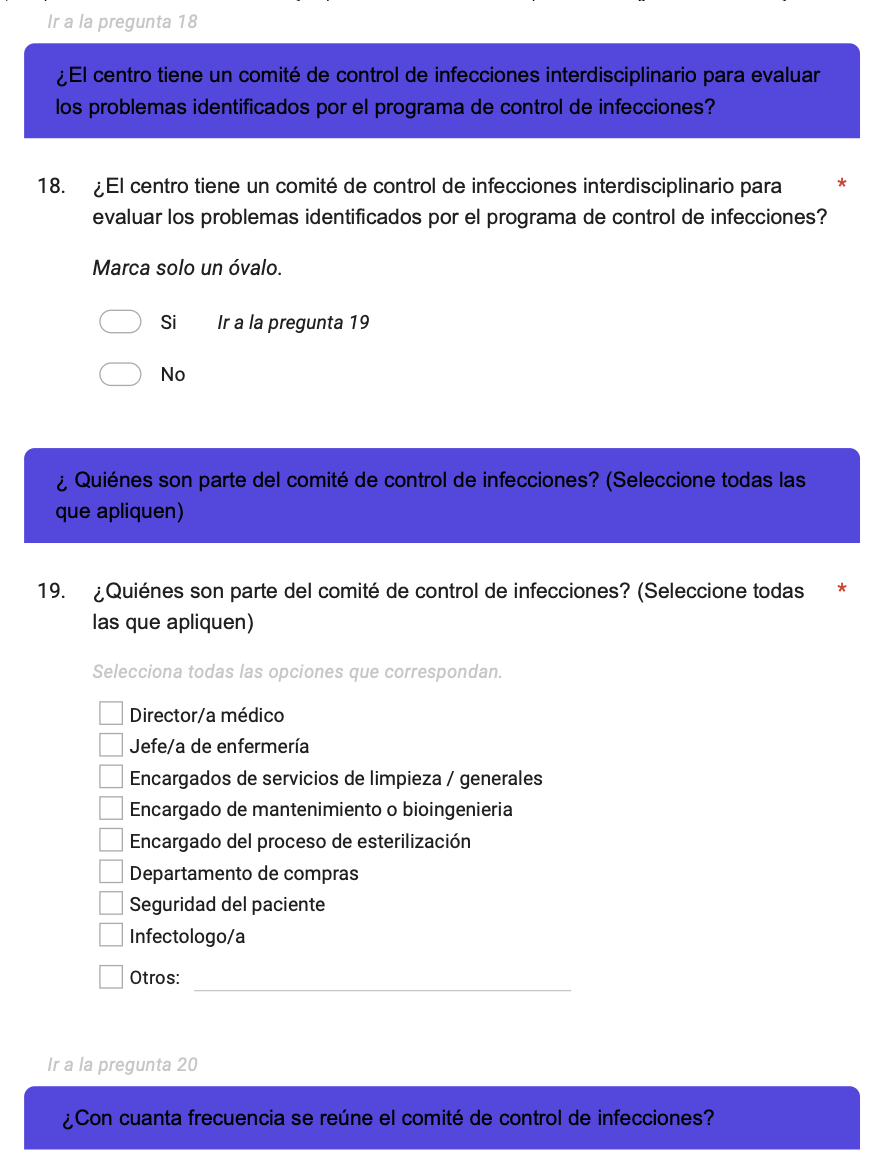

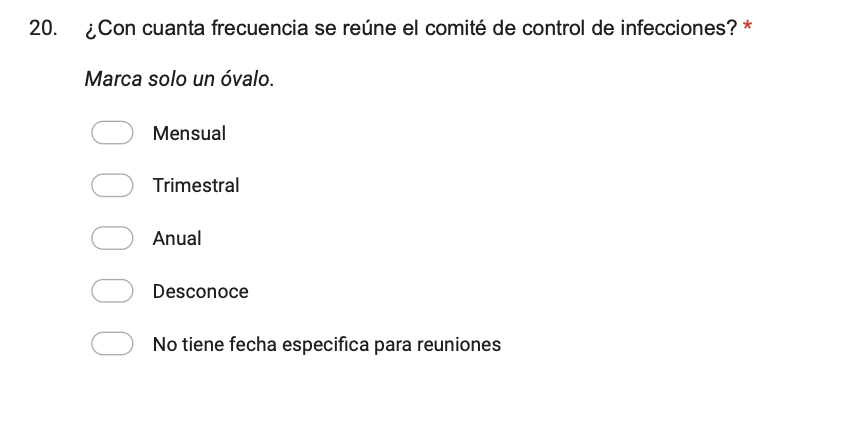

Supplement: Castillo et al. supplementary material [file S2732494X26107888sup001.docx]
